# Sources of Life Strengths Appraisal Scale: A Multidimensional Approach to Assessing Older Adults' Perceived Sources of Life Strengths

**DOI:** 10.1155/2014/783637

**Published:** 2014-03-09

**Authors:** Prem S. Fry, Dominique L. Debats

**Affiliations:** ^1^Graduate Psychology Program, Trinity Western University, 7600 Glover Road, Langley, BC, Canada V2Y 1Y1; ^2^Private Practice, 9711 RS 2E4 Groningen, The Netherlands

## Abstract

Both cognitive and psychosocial theories of adult development stress the fundamental role of older adults' appraisals of the diverse sources of cognitive and social-emotional strengths. This study reports the development of a new self-appraisal measure that incorporates key theoretical dimensions of internal and external sources of life strengths, as identified in the gerontological literature. Using a pilot study sample and three other independent samples to examine older adults' appraisals of their sources of life strengths which helped them in their daily functioning and to combat life challenges, adversity, and losses, a psychometric instrument having appropriate reliability and validity properties was developed. A 24-month followup of a randomly selected sample confirmed that the nine-scale appraisal measure (SLSAS) is a promising instrument for appraising older adults' sources of life strengths in dealing with stresses of daily life's functioning and also a robust measure for predicting outcomes of resilience, autonomy, and well-being for this age group. A unique strength of the appraisal instrument is its critically relevant features of brevity, simplicity of language, and ease of administration to frail older adults.

*Dedicated to the memory of Shanta Khurana whose assistance in the pilot work for the study was invaluable*

## 1. Introduction

Given the resurgence of interest in the field of positive psychology [1], both gerontologists (e.g., [2, 3]) and social psychologists (e.g., [4]) concur that older adults' appraisals of their life strengths are at the very basis of older adults' identity development and contribute significantly to their perceptions of empowerment.

In recent years, an approach known as the “life strengths perspective” has emerged both in the theoretical literature (e.g., [5]) and in the practice literature as extended to the treatment and clinical approach to older adults ([6, 7]). The life strengths perspective recognizes that, even in the most difficult circumstances, there is reciprocity between older adults' personally constructed views of reality and their social environment (see [8, 9]). Another conceptual framework that is related to the life strengths perspective, in collaboration with the resilience theory perspective, is Maslow's self-actualization perspective [10]. It is reasoned that internal and external sources of life strengths that older adults identify within themselves reinforce personal identity and actualization, factors which are the cornerstone of resilience in older adults (see [5, 7]). The life strengths perspective represents a paradigm shift in clinical practice wherein practitioners focus on assessing and magnifying the worth, dignity, and uniqueness of older adults by helping them identify strengths and resources that facilitate and promote the actualization and achievement of client goals and plans for success [11] in contrast to highlighting disabilities.

Accordingly, what is urgently needed by professionals and by older adults themselves is a* self-report assessment scale* that enables older clients to identify, appraise, and take stock of various aspects of the sources of social, emotional, and cognitive strengths that may assist them in facing both problems of daily life functioning and also life's challenges, crises, and struggles (see [11–14]). In the present research we describe various stages in the development of a psychometrically valid instrument targeted to assess older adults' appraisals of important dimensions of internal and external sources of strengths and capabilities. We subsequently demonstrate through a 24-month followup of a selected group of study participants, how individuals' self-assessments of their various sources of life strengths are a robust measure for predicting outcomes of resilience as defined previously by authors of earlier standardized scales of well-being in terms of constructs such as perceived challenge, control, and engagement [15] and in terms of perseverance, equanimity, meaningfulness, self-reliance, and existential aloneness [16].

## 2. Conceptual Framework for the Study of Life Strengths

Our assumptions and hypotheses concerning older adults' appraisals of the sources of life strengths are embedded within three distinct conceptual frameworks which we sum up as follows.

1. *The existential theory framework* which argues that the search for new and emerging sources of life strengths is continual throughout life but greatly accelerated in late life as individuals move toward seeking and defining their meaning for life and achieving some measure of self-transcendence over the stress and pain of physical and emotional losses [17–20]. Hoffman [21] suggests that “the attainment of meaning is one of the most central aspects of human existence and necessary to address in existential therapy” (p. 49). From the perspective of existential theorists, individuals' appraisals of the sources of life strengths will vary in terms of the personal maturity and depth of meaning they perceive themselves to have achieved. These are assumed to enhance both physical and psychological well-being and to predict resilience and autonomy in overcoming adversity (see [17, 22, 23]).
2. *The social-cognitive framework *originally proposed by Bandura [4] stresses the primacy of the human agency and the acquisition of empowerment through self-efficacy beliefs and beliefs about control. This perspective embraces a broad ecological view of human growth which argues that individuals' strength and persistence in the face of challenges and adversity come from their self-efficacy beliefs and positive perceptions of their own knowledge, competence, and expertise to resolve problems and dilemmas. A number of empirical studies in the aging literature (e.g., [24–26]) show how self-efficacy beliefs provide the foundations for higher levels of life satisfaction and self-esteem (see [27]) and are sound predictors of older adults' psychological resilience.
3. *The psychosocial framework* posits that thematic strengths in old age functioning are directly linked to unique socioeconomic challenges that older adults encounter, which are embedded in the changing social, economic, and political structures of Western society [28]. This framework is closely tied in with the psychological framework of successful aging (e.g., [29, 30]) to include older adults' personal needs to achieve maximum optimization and resilience in late-life functioning within the social, economic, and political structures of society [31], and to pursue goals in late life that allow for autonomy and continuity of personal commitments and aspirations that were developed in earlier life stages [32].

Throughout the literature (starting with Maslow [10] and continuing into the current literature detailed in Ungar [9] and Vaillant [14], there is agreement that the search for various sources of life strengths is embedded in multidimensional contexts and contributes significantly to individuals' appraisals of their self-worth and sense of well-being. However, it is recognized that individuals might differ in the extent to which they appraise internal sources of strengths, as distinguished from external sources of strength, more positively or vice versa in facing life stressors (see [13]).

### 2.1. Sources and Determinants of Life Strengths and Their Relationship to Constructs of Vulnerability and Resilience

Based on theoretical dimensions of sources of life strengths we reviewed in the existential, social-cognitive, and psychosocial frameworks, it is reasonable to define the global construct of life strengths as a constellation of inner resources including skills, habits, beliefs, values, goals, behaviors, commitments, and expert knowledge, as well as the ability and resourcefulness to draw on resources in the external environment (e.g., social support and monetary gains) in order to adapt to changing circumstances. These agentic factors are a powerful source of personal strength and protective power and contribute to resilience which is conceptualized in terms of protective factors within the individual. Resilience defined as a “defense mechanism which enables people to thrive in the face of adversity” [33] is instrumental in both the individual's recovery and flourishing after the recovery [34]. However, resilience is not necessarily innate to the individual, but is largely contingent on the individual having sufficient inner and external resources that capacitate the person to endure, bounce back, and grow in the midst of adversities and existential anxieties [34, 35]. The underlying research and science-based assumption is that the adaptive processes underlying resilience development are intrinsically embedded in the vast expanse of life strengths that individuals have in their repertoire of learning and development. The greater the number of sources of life strengths, the greater the number of protective factors within the individual leading to higher levels of resilience and related positive health outcomes in the face of significant challenge, adversity, and stressful circumstances [36, 37]. Recently, Ungar and his associates [38, 39] have broadened resilience to include ecological and cultural factors. They define resilience as the development and application of science-based knowledge pertaining to positive development, positive adjustment, and thriving across the life-span. Thus, the construct of sources of life strengths is intrinsically linked to the concept of resilience in that they are acknowledged to be both intrapersonal and process-oriented and have a reciprocal relationship. Briefly stated, the “sources of life strengths” construct is a multidimensional and multifaceted dynamic construct assumed to have a reciprocal relationship with a number of other primary constructs, such as resilience and persistence, and other internal sources of self-regulatory controls such as self-efficacy, maturity, and sense of control, and external sources (such as social support and socioeconomic resources) that may impact positively or negatively on social, emotional, and intellectual well-being [40–42].

There is agreement that, while each framework we have considered in our review of the gerontological literature emphasizes a different set of independent structures and pathways for the emergence of life strengths, we postulate that collectively they all contribute to the maintenance and enhancement of resilience. Thus, our major assumption for the instrument development was the potential for plasticity in older adults. In developing our instrument on older adults' perceived sources of life strengths we worked from the basic assumption that older adults' appraisals of their internal strengths are protective mechanisms that are robust predictors of dispositional resilience in late life functioning [43].

A search of the literature revealed that, although there is a range of existing screening instruments that assess individuals' perceptions of their life strengths, each measure focuses narrowly on one single dimension only, for example, purpose for life (e.g., [22]), global self-efficacy beliefs (e.g., [44]), sense of control measure (e.g., [45]), and social support (e.g., [46]). Although these measures are widely accepted as screening instruments, their usefulness with respect to assessing older adults' sources of strengths is limited for a number of reasons. First, the measures, as noted, are not validated exclusively on samples of older adults. Second, their usefulness as a general measure of sources of life strengths of older adults is limited in that the focus is on one single (unitary) dimension, structure, or source of life strengths. Third, many items of the self-report measures are not easily understood by older adults themselves, and frequently must be interpreted to them by professionals. Accordingly, there is a strongly felt need for the development and validation of one easily comprehensible and reliable self-report instrument that takes into account multidimensional and multifaceted internal and external sources of life strengths of older adults.

## 3. Overall Objectives of the Study

Specific aims of the current study were fourfold: (1) to design an instrument “*sources of life strengths appraisal scale (SLSAS)” *specifically aimed at identifying a number of relevant cognitive and social-emotional dimensions of life strengths critical to late-life functioning; (2) to establish evidence of its factorial validity; (3) to assess the reliability of this instrument; and (4) to present evidence of the concurrent and predictive validity of the SLSAS measure with respect to ascertaining resilience potential of the study participants.

## 4. Method

### 4.1. Sample Pool for Study

The sample pool for this study was comprised of a total of 540 older adults, including a sample of 110 randomly selected volunteers for the pilot phase of the study, and another 430 older adults who were subsequently recruited as three independent samples (sample 1,  *n* = 120; sample 2, *n* = 120; sample 3, *n* = 190) as used in the study. All participants in the pilot sample and three study samples were between the ages of 65 to 89 years residing in three large cities and semirural communities in Southern Alberta and British Columbia. Participants were recruited from a variety of civic, social, recreational, and semi-institutional settings and consisted of an almost equal number of females (51.3%) and males (48.7%). The mean age was 72.6 years, with all years between 65 and 89 represented. Participants were predominantly Caucasian/White (78.7%). Level of education ranged between a minimum of a junior high school certificate (17%) to a university degree (21%). A majority of the adults (42%) had a high school level education. At the time of recruitment of participants, 36% reported that they were in “excellent” health, 40% in “fair to good” health, and 24% in “poor” health. Participants were from all walks of life, ranging from being “unskilled” to “blue collar” workers, to “white collar professionals”, and “competent executives”.

Our systematic plan was to examine the concurrent validity of the SLSAS measure by comparing various SLSAS factor scores with other existing measures of mental health, depression, or other forms of vulnerability. In accordance with this plan, we administered a number of other test measures against which we could assess the concurrent validity of the SLSAS measure.

### 4.2. Measures

All study samples were administered the following measures that are well-reputed standardized measures developed by earlier researchers. They were included in a counterbalanced order in the questionnaire booklet. Participants were given the choice to complete the measures at their own pace, at their permanent home, or in their place of temporary residence (such as assisted living quarters or group homes). Participants arranged to return the materials at their earliest convenience to a designated research assistant. Participants who did not return the response materials within the first two weeks were recontacted by the research assistant who offered to give any additional help, if needed, for pickup of the materials, or for any difficulty in responding to the research questionnaires.

*(1) Sources of Life Strengths Measure (SLSAS).* (For a description of this measure and its development, see details in Section 4.) 

*(2) Sense of Control [45]*. In this standardized measure, the sense of control is operationalized with two dimensions: personal mastery and personal constraints. Following Lachman and Weaver [45], we selected two items measuring Personal mastery: “I can do just about anything I really set my mind to” and “When I really want to do something, I usually find a way to succeed at it.” “Perceived constraints” were measured using four items (e.g., “Other people determine most of what I can do to change many of the important things in my life,” “I often feel helpless in dealing with problems in my life”). A 4-point rating scale (ranging from 1 = a lot to 4 = not at all) was used for both dimensions. Using this scale, we derived a global score of internal control. Cronbach's alphas for respondents in the three samples of the present study ranged from .73 to .76 and from 70 to .73 for men and women, respectively. With respect to this measure of control, our hypothesis was that the SLSAS measures would correlate positively with high internal control. 

*(3) Measure of Self-Esteem (SEI: [47])*. This inventory was used to measure self-esteem as a global and stable disposition. The inventory has 10 items, 5 positively keyed and 5 negatively keyed. Each item is rated on a 4-point Likert type scale. Cronbach's alphas for respondents in the three samples of the present study ranged from .83 to .85 and from .79 to .82 for men and women, respectively. With respect to the measure of self-esteem, our hypothesis was that the SLSAS measure would correlate positively with high self-esteem on the SEI. 

*(4) Life Satisfaction Was Assessed by Means of the Five-Item Satisfaction with Life Scale (SLS: [48])*. Each item is rated on a 5-point Likert type scale, yielding a score range of 5 to 25. The measure assumes that participants compare their current circumstances against subjective standards to arrive at a global appraisal of life satisfaction (e.g., “In most ways my life is close to ideal”). Cronbach's alphas for respondents in the three samples of the present study ranged from .81 to .86 and from 79 to .82 for men and women, respectively. With respect to the measure of life satisfaction (SLS), our hypothesis was that the SLSAS measure would correlate positively with high self-esteem on the SLS. 

*(5) Measure of Vulnerability [49]*. This is a 12-item screening test to measure older adults' state of vulnerability in terms of anxiety, dependence, helplessness, and rejection. In the present study, each item was scored on a 4-point Likert type scale with scores ranging between 12 and 48. Responses were summed to give a total vulnerability score. In the context of this scale, vulnerability is conceptualized as being the opposite of resilience. Vulnerable individuals are characterized as being low in resilience and reserve capacity. Psychological vulnerability from the perspective of this scale is viewed as morbidity, depression, and psychological distress. Cronbach's alphas for respondents in the three samples of the present study ranged from .80 to .86 and from .79 to .83 for men and women, respectively. Our hypothesis with respect to this measure of vulnerability was that the SLSAS measure would correlate negatively with vulnerability dimensions of stress, anxiety, and depression. 

*(6) Depression*. Depression was measured by the Zung self-rating depression scale (SDS: [50]) covering depressive symptoms relating to somatic, psychological, psychomotor, and mood areas. A total depression score was calculated for each respondent by summing scores in the four domains. Cronbach's alphas for respondents in the three samples of the present study ranged from .80 to .84 and from .78 to .81 for men and women, respectively. With respect to the measure of depression (SDS), our hypothesis was that the SLSAS measure would correlate negatively with the depression measure on the SDS. 

### 4.3. Two Outcome Measures Administered at 24-Month Longitudinal Followup of Respondents Procedures Related to Predictive and Concurrent Validity of SLSAS

Additionally, we also planned on conducting a 24-month (two years) followup of respondents from the time of baseline assessments to obtain evidence of the predictive validity of the SLSAS with respect to ascertaining resilience potential of the study participants.

Thus, following baseline assessment of the SLSAS, a group of 150 respondents were randomly selected from the overall sample of 540 older adults for a 24-month followup. The purpose of the followup was to assess the predictive validity of the SLSAS with respect to outcome measures of resilience. The randomly selected sample of 150, who at the time of baseline agreed to a 24-month followup, was administered with the following two additional measures of resilience.

*(1) Dispositional Resilience Scale* (DRS: [15]). This measure is composed of 45 items with 15 items each assessing* commitment to living *(e.g.,most days life is interesting and exciting for me),* control* and* focused engagement *(e.g.,planning ahead can help me avoid most future problems), and* challenge* (e.g., changes in routine are interesting to me). The DRS was originally developed as a measure of hardiness. In the context of this scale, resilience in later adulthood is a form of hardiness or mindset that facilitates recovery from risk and adversity (see [35, 43]). Those who typify psychological hardiness espouse a commitment to living; they enjoy challenge and believe that change rather than stability is normal. Also they manifest a pervasive belief that they can and will respond effectively under stress [51]. Internal consistency of responses as measured by Cronbach's alpha was .82 and .83 for men and women, respectively, in our sample.

Our hypothesis was that the greater the number of internal and external sources of life strengths that are identified by the SLSAS scale, the stronger the association with the three domains of resilience in the DRS, at the time of the 24-month followup. Internal consistency of responses as measured by Cronbach's alpha for the DRS* Scale* was .79 and .82 for men and women, respectively. 

(2)* The Resilience Scale* [16]. We used the 50-item version of the resilience scale as originally constructed [16] to measure the potential for resilience. The construct of resilience is defined in terms of five characteristics:* perseverance* (act of persistence despite adversity or discouragement),* equanimity* (balanced perspective of life and experiences that moderate the extreme responses to adversity),* meaningfulness *(the realization that life has a purpose and recognition that there is always something for which to live),* self-reliance* (the ability to believe in one's self and to rely on one's personal strengths and capabilities in the face of adverse circumstances to guide one's actions), and* existential aloneness* (the realization that each person is unique and that, while some experiences can be shared, others must be faced alone). These five characteristics form the conceptual foundation for the* resilience scale* (TRS) [16]. Internal consistency of responses as measured by Cronbach's alpha for the* resilience scale* was .82 and .83 for men and women, respectively. 

Our hypothesis was that the greater the number of internal and external sources of life strengths that are identified by the SLSAS scale, the stronger the association with the five components of resilience as detailed in the* resilience scale *(TRS) [16] at the time of the 24-month followup.

## 5. Construction of the Preliminary SLSAS

### 5.1. Use of Focus Groups for Generating Items and Item Selection

We resorted to focus group methodology that other recent researchers have found particularly useful for the purpose of generating items for preliminary scale development and for ensuring that a diversity of theoretical constructs and dimensions are considered in the scale development (see [52]). There were 48 focus group members assigned in equal numbers to four subgroups of 12 individuals (6 men and 6 women). Each subgroup included both lay persons and professionals. Assisted by two graduate student researchers who searched the psychology literature, each focus group participated in four discussion sessions of approximately 1- to 1.25-hour duration. They brain-stormed and explored the core focus and key concepts of internal and external sources of life strengths as identified in the literature, and delineated several constructs relevant to the major themes. Collectively, the focus group teams drew attention to the major theoretical constructs that might serve as the bases for generating items for the current scale development. Key dimensions that were suggested and discussed for item generation included the following areas of focus:

1. factors conducive to coping with normative losses in old age (see [31]),
2. factors conducive to personal resilience (i.e., factors conducive to recovery from risk and adversity and factors conducive to the maintenance of developmental capacities in the face of cumulating threat and challenge);
3. factors conducive to development of social identity and social well-being (i.e., factors conducive to the attainment of stability and structure in one's social life) (see [14]); factors conducive to the expansion of the self and/or self-knowledge (i.e., perception of continuity in one's life, provision of meaning in life, perceptions of positive self-regard, a sense that life is purposeful, a sense of continuing growth, and a general sense of life satisfaction) [30, 53]; and actualization of self and life goals [10];
4. factors conducive to a sense of altruism and morality (i.e., factors conducive to the ability to follow inner convictions and to live life in the context of giving, sharing, and receiving) [54];
5. factors conducive to a sense of affiliation and belongingness (i.e., factors conducive to perceptions of having good quality relationships with family and others) [10, 12];
6. factors conducive to a sense of stimulation, challenge, and excitement/enjoyment (i.e., factors conducive to the provision of novelty and exciting and meaningful experiences);
7. factors conducive to a sense of accomplishment and competence (i.e., having perceptions of goals in life);
8. factors conducive to a sense of social superiority by comparison and competition with others (i.e., reflection of one's achievement over others and a sense of victory) (see [26]);
9. factors conducive to a sense of power, influence, and control (i.e., the provision of opportunity to exert effect on others' lives) [4] (see also discussion by [5, 12]).

Each of the focus group sessions lasted on average for 1 to 1.25 hours and the sessions collectively generated an initial multiple item pool of 85 items that tapped individuals' perceptions of sources of life strengths that are valued most in different situations of stress, challenge, adversity, success, victory, and flourishing. Five to 7 items operationalizing each of the selected key theoretically relevant dimensions were generated by members of the focus group, based on their understanding of key themes drawn from the literature on developmental aging that were suggested to them by the graduate research assistants. The 85 items were submitted to three contents experts (a recently retired university professor, one experienced social worker, and one geriatric practitioner) for judging the importance of each item (YES-NO) with respect to its relevance to an identifiable key construct or a key dimension of strengths identified in the literature. All 85 items were judged to be of importance. A satisfactory level of interrater agreement was obtained if two of the three raters agreed on the classification of the item to a construct domain that had been identified during item generation.

All components judged by focus group experts as being important and relevant to the “sources of internal strengths” construct were picked out and expanded into complete sentences that allowed respondent(s) to rate the extent to which they believe the item component “*as being a source of strength for me during times of difficulty, or generally contributing to my overall sense of psychological well-being”* was “very important” = 5 to “not important” = 1.

Based on interrater agreement ratings, 60 items out of the original 85 items were judged to be “important” and were retained. The remaining 25 items were discarded. Based on the preceding item selection procedures, the preliminary SLSAS was defined as a 60-item instrument and subsequently administered to one pilot group of 110 members and subsequently to three sample groups (sample 1, *n* = 120; sample 2,  *n* = 120; sample 3, *n* = 190) for further reliability.

## 6. Results

### 6.1. Instrument Evaluation on a Pilot Group

The condition of the data matrix was evaluated, first, by testing the adequacy of the sample correlation matrix on a pilot group of 110 persons for the 60 items of our scale by using the Kaiser-Meyer Olin (KMO) measure for sampling adequacy [55]. This was calculated to be .90, attesting that the sample size was large enough to evaluate the factor structure [55]. Second, we tested the factorability of the data set using Bartlett's test of sphericity, *χ* ^2^  (528/3 = 3711.72) that was significant at the .0001 level indicating further that the data matrix approximates an identity matrix and that the factor model was appropriate for a principal components analysis [56].

### 6.2. Factorial Validity

To empirically test the validity of the SLSAS items that were developed, two separate factor analyses were conducted (see Table 1).

#### 6.2.1. Primary Analysis: Exploratory Factor Analysis (EFA) (See EFA, Table 1)

A preliminary (exploratory) factor analysis using the method of factorial components with Varimax rotation was performed to determine the factor structure of the preliminary SLSAS items. Data used in the exploratory factor analysis were obtained from the administration of the preliminary SLSAS items to 240 respondents (samples 1 and 2 combined) from the total sample (see Table 1). Factors that yielded an eigenvalue greater than one were deemed to merit further consideration. Because we desired a relatively brief instrument, we used the loading criteria suggested by Stevens [57]. Accordingly, we decided that there must be a minimum of five items, with factor loadings of .40 or higher within a factor, for the factor to be considered for interpretation. Furthermore, it was decided that, if an item loaded on more than one factor, then the factor with the highest loadings would be selected.

Thirteen component factors with eigenvalues greater than one emerged from the preliminary factor analysis (EFA) (see Table 1). We agreed that, in addition to the “eigenvalue-one” criteria, we consider two other criteria to decide how many component factors to retain. First, we examined the scree plot [58] and observed the break between components with large eigenvalues and those with small eigenvalues (but not less than one). In our data, we observed a major and notable break which occurred at 10 components that accounted for a cumulative percentage of 70% of the explained variance that is deemed appropriate for initial scale development (see [57, 59]). This 10-factor solution, which could be considered as a relatively conservative representation of the data, was rotated to a Varimax criterion for further interpretation. The first component had an explained variance of 38% followed by five more accounting for a cumulative variance of 20% and the last four providing a cumulative variance of 12% (see footnote in Table 1). Based on the relative strengths of factors as determined by the percentage of the explained variance, the definition and labeling of the first six factors was as follows: (1) personal commitment; (2) self-efficacy beliefs; (3) personal maturity; (4) attitudes toward life; (5) continuity in mid-life roles; (6) personal achievements. These six factors accounted for 54% of the total cumulative variance, with the remaining variance of 16% accounted for by the four factors that we labeled and defined as follows: (7) informal social support; (8) monetary status; (9) personal traits; and (10) self-care routines.

#### 6.2.2. Second Analysis: Principal Components Factor Analysis (PCA) (See PCA, Table 1)

A second principal component factor analysis with Varimax rotation was performed on the data derived from the administration of the preliminary SLSAS to 190 new respondents. It is important to note that these 190 individuals were new respondents who had not participated in any of the earlier stages of item generation, nor had been involved in any reliability assessment of the SLSAM research. Therefore, they had no familiarity with the SLSAM items or subscales that had been generated to this point. The purpose of the second factor analysis was to replicate the factor structure identified in the primary (exploratory) factor analysis (EFA) and to estimate the relative independence of the established factors in the context of a new independent study sample. Thus, the second principal component analysis was intended to serve a confirmatory function (PCA). In this analysis, the first component had an explained variance of 32% followed by five more accounting for a cumulative variance of 23% and the last three providing a cumulative variance of 15% (total explained variance = 70%) (see footnote in Table 1). Results of the PCA showed nine factors with factor loadings of .40 or higher (see Table 1: PCA).

With the exception of the personal achievement factor, the factor structures that emerged in the PCA strongly confirmed the strength of the original factor structures reported in the exploratory factor analysis (EFA). The total amount of variance explained by the six most salient factors in the PCA was 52%, compared with 54% in the EFA. In both factor analyses, the communalities ranged from .77 to .25 and from .74 to .22, respectively, indicating that items shared a reasonable amount of variance with all other items (see [60]). The finding of an overall close correspondence between the factors identified in the two sets of factorial analysis provides robust evidence that the factor structure within the SLSAS items is relatively stable across samples.

### 6.3. Composite Description of the SLSAS

Based on the findings of the two independent factor analyses, the structures of the SLSAS were described as follows.

Internal sources of life strength were (1)* personal self-efficacy* (five items) which includes beliefs of one's competence to handle life's situations and motivation to overcome problems; (2)* personal maturity* (five items) which includes such components as meaning for life, purposes and goals for living, a sense of spirituality, acceptance of one's limitations and acceptance of negative consequences; (3)* personal traits* (five items) which includes dispositional characteristics such as courage, self-confidence, sense of personal responsibility, and accountability; (4)* self-care routines* (five items) such as habits of diligence, persistence and determination in task performance, sound dietary habits, and other habits such as physical exercise, devotional prayer, and relaxation; (5)* personal commitments *(five items) which include the individual's commitments to make a positive impact on others, or to support and advocate causes in the public interest; (6)* attitudes toward life *(five items) which include elements such as the individual's positive outlook on life, viewing life to be a challenge and adventure, and believing in the goodness of human nature.

External sources of life strengths were (7)* social support: informal sources* (five items) representing support coming from intimate relationships, friends, and associates; (8)* monetary status* (five items) which includes monetary advantage such as a steady income in terms of retirement and old age security benefits, financial investments, and inherited assets; (9)* continuity in mid-life roles* (five items) which refers to opportunities available to continue in employment after retirement age, opportunities to preserve and sustain long-term relationships from the mid-life years, and opportunities to gain recognition for new and past activity and functions.

It should be noted that the factor of “personal achievement” ratified in the first EFA did not get commensurate factor loadings in the second confirmatory PCA, and accordingly it was dropped from further consideration in the instrument development (specimen items from each structure that were retained in the final version of the 45-item SLSAS are included in the Appendix).

### 6.4. Reliability Assessments of the SLSAS Measure

In order to investigate the psychometric quality of the SLSAS scales that were created to correspond with factors identified in the preliminary factor analyses and cross-validated with another confirmatory factor analysis, we pursued a number of other preliminary but vitally important steps.* First,* following Nunnally [61], we set a predetermined standard for internal reliability (Cronbach's *α*) at .70. Cronbach's alphas were calculated and these ranged from .71 to .81 for ten scales that were derived from the EFA analysis. Cronbach's alphas ranging from .82 to .89 were observed for nine scales derived from the PCA, indicating moderate to very good internal reliability appropriate for a relatively brief screening instrument [62].

*Second*, we examined the correlations among the SLSAS subscales themselves (Table 2).

Findings showed that they were moderately correlated, with correlations ranging from .12 to .28. The mean intercorrelation was .21. This suggests that these scales, despite the potential for some overlap, are tapping relatively independent appraisal dimensions.

*Third*, we conducted an inspection of item-total correlations of all the items, factor matrix, and communalities. Out of the original 60 items, only 45 items showing interitem correlations ranging from .15 to .20, and subscale correlations ranging from .18 to .22, were retained as being acceptable [63]. As a final step, the reliability of the final SLSAS was assessed using internal consistency estimates (Cronbach's alpha) for the separate samples. With a few exceptions, alphas for six of the* “internal sources of strength”* scales and three of the* “external sources of strength” *scales were good, ranging from .73 to .94. Cronbach's alphas for three scales were lower than our predetermined criterion of .70, and therefore these scales (personal achievement, social support from formal sources, and monetary stability) were dropped from further analysis.

Test-retest reliability over a 6-month interval was fully acceptable with test-retest reliability coefficients ranging from .79 to .82 for the nine subscales. Internal consistency of responses ranged from *α* = .79 to *α* = .85 (*further raw data details about item-total correlations, factor matrix, communalities, and retest reliability data are available from the authors*).

Examination of the mean scores showed that, in most of the scales, the mean scores were approximately in the middle range, with reasonably high mean appraisal scores assigned to personal commitments, personal self-efficacy beliefs, personal maturity, personal traits, self-care routines, life attitudes, social support (informal), monetary status, and continuity in mid-life roles.

Based on the results of the preceding reliability procedures and the results of two factor analyses (EFA and PCA), more specific definitions were arrived at for the nine scales of the final 45-item version of the SLSAS. The Principal components factor analysis of the SLSAS reported that the factors also belong to a latent construct of the overall sources of life strengths and this score can be achieved by a summation of the nine subscale scores. Cronbach's alpha for the summed scale is .81, now being a summated rating scale employing a 5-point response format (ranging from* Not At All Important* to* Very Important)*. In its final form, the SLSAS is scored by summing the responses to all 45 items, with subscale scores obtained by summing the responses to five items within each subscale (i.e., structure).

### 6.5. Further Evidence of Concurrent Validity of SLSAS

Further evidence of concurrent validity for the SLSAS was obtained by examining the correlations between the SLSAS scales and other related psychological variables, notably variables of internal control, self-esteem, life satisfaction, vulnerability, and depression. Examination of the correlations of the SLSAS factor structures with various measures (see Table 3) shows that almost all of the correlations are at a significant level (ranging from .21 to .69).

Consistent with our hypothesis, we observed a positive correlation between the subscale scores on the SLSAS and variables of internal control, self-esteem, and life-satisfaction. Conversely, consistent with our hypotheses, we observed a negative association between most of the subscale scores on the SLSAS and variables of vulnerability and depression. These findings provide further evidence of the concurrent validity of the SLSAS life strength structures corresponding with key theoretical dimensions of mental health fitness (e.g., internal control, self-esteem, and life satisfaction) as underscored in the recent gerontological literature. Also, the negative association of the SLSAS subscales and the measures of vulnerability and depression is consistent with the trends reported in the gerontological literature.

### 6.6. SLSAS as a Predictor of Outcomes Measures of Dispositional Resilience (Control, Challenge, and Commitment) and Resilience Characteristics (Perseverance, Equanimity, Meaningfulness, Self-Reliance, and Existential Aloneness) at the 24-Month Followup

The predictive validity of the SLSAS measure was further established by conducting a 24-month follow-up study of 150 randomly selected respondents, commencing from the time of baseline administration of the final SLSAS measure and two measures of resilience: (1) Bartone et al.'s measure of dispositional resilience (DRS) and (2) the resilience scale ([16] further modified in 2009), These instruments were readministered at the time of the 24-month followup. Correlation coefficients of the SLSAS subscales with three subscales of dispositional resilience (DRS) (i.e., perceived challenge, control processing, and commitment to Living) were examined (Table 4). Similarly, correlation coefficients of the SLSAS nine subscales with five subscales of the Wagnild and Young [16] resilience scale (further modified in 2009) (i.e., perseverance; equanimity; meaningfulness; self-reliance; existential aloneness) were examined (Table 5).

The results clearly demonstrate that there were robust correlations (all in the hypothesized direction) between the nine SLSAS subscales and the three subscales and the total of the dispositional resilience scale (with correlations ranging between .21 and .69) (see Table 4). Similarly, there are robust correlations (all in the hypothesized direction) between the five dimensions of the Wagnild and Young [16] resilience scale and nine SLSAS subscales (i.e., correlations ranging from .41 to .69).

Subsequent multiple regression analyses conducted for predictors of resilience showed that, after controlling for demographic variables, subscales of personal self-efficacy, personal commitments, attitudes toward life, and continuity in-mid-life roles in the SLSAS were the most significant predictors of resilience (as defined in the two resilience scales that were administered in the 24-month followup), accounting for a significant proportion of the variance (76% and 79%, resp.). These data attest further to the fact that the SLSAS has the potential to predict outcomes of challenge, control, and commitment as defined in the* dispositional resilience scale*. Similarly, the SLSAS has the potential to predict outcomes of perseverance, equanimity, self-reliance meaningfulness and existential aloneness as defined in* the resilience scale *[16].

## 7. Discussion

### 7.1. Strengths of the SLSAS Measure

In terms of unique strengths, the SLSAS is among the first of validated measures to provide support for the notion that what keeps a majority of older individuals strong in the face of both daily life stresses and obstacles and adversity is a balance of perceived life strength resources, both internal and external. The major strengths of the instrument can be observed in terms of the multidimensional internal and external resources it taps, and its capability to integrate, as parsimoniously as possible, several useful constructs that have hitherto been embedded in a multiplicity of single construct assessment scales and measures. While the present study is cross-sectional and causal directionality, therefore, cannot be imputed, our results from the SLSAS measure show, at the least, a significant relationship between various dimensions of the “life strengths” construct, such as personal self-efficacy beliefs, personal maturity, personal traits, self-care routines, personal commitment, personal achievements, positive attitudes toward life, social support, monetary status, and continuity in mid-life roles, and older adults' subsequent self-appraisals of resilience/hardiness, internal control, self-esteem, and life satisfaction when encountering situations of stern challenges, threat and uncertainty.

The final version of the 45-item SLSAS is derived from two rounds of exploratory factor analysis and principal factor analysis centered on nine appraisal dimensions (structures) comprised of 5 items per structure. It should be noted that the SLSAS measure was validated on four samples of older adults (one nonrandom pilot sample and three randomly selected samples) ranging in age from 65 to 87 years. Within the predetermined scope of the present study, we were able to establish the concurrent validity by assessing the association of the structures of the SLSAS with other related measures of self-esteem, life satisfaction, vulnerability, and depression. Additionally, it should be noted that concurrent validity of the instrument was based on a postbaseline followup of 25% of individuals drawn randomly from the original Pilot sample and samples 1, 2, and 3. Another unique feature of the SLSAS is that we were able to establish the predictive validity of the SLSAS measure over a 24-month followup, specifically with respect to predicting older adults' resilience as measured by other well-reputed standardized scales of resilience. It is important to note that the sample of 540 older participants, involved in various phases of the validity and reliability procedures, is representative of individuals who reported a range of health statuses, a range of educational levels, and a range of occupational backgrounds and financial status. The SLSAM was thus validated on a sample of older adults appropriately representative of individuals from various walks of life.

Another significant feature of the instrument is that it is brief, requiring, on average, about 10 to 12 min. response time as opposed to a multiplicity of single dimension measures which are commonly used to assess the same nine factors, and which are reported to be extremely time-consuming for the average older adult. Thus, it has ease of administration to frail older adults who have trouble responding to lengthy questionnaires frequently precipitating fatigue and unreliability in response ratings (see [64]).

To conclude, the SLSAS instrument meets stringent criteria of reliability, construct, and predictive validity essential to the development of new screening instruments (see [62] for discussion of practical and theoretical considerations in developing brief screening instruments). The SLSAS is a valid omnibus measure of older adults' life strengths described variously as a constellation of beliefs about the self, one's values and commitments, one's perceptions of competence, abilities and controls, and one's perspective of social domains outside the self.

### 7.2. Implications and Applications of the SLSAS for Counseling Practice with Older Adults

As a relatively brief assessment instrument, the SLSAS maybe useful to geriatric professionals and health professionals who wish to obtain a multidimensional picture or profile of their elderly clients' highly valued sources of strengths with the aim of predicting their future psychological well-being and resilience capacity. In short, the SLSAS instrument offers counseling psychologists and other geriatric service providers a reliable and valid, but also sensitive, approach to assessing older adults' self-appraisals of a diversity of internal and external sources of life strengths. This is important client information for counseling psychologists to consider. Mounting evidence points to the importance of older adults' perceived personal strengths as a valid indicator of their overall resilience with respect to psychological health and well-being [9, 65]. Also there is increasing evidence suggesting that older adults' self-perceptions and self-appraisals of their life strengths and sources of strength have an important influence on their objective health and well-being, with poor appraisals and negative perceptions of internal strengths leading to greater helplessness and loss of control, and positive appraisals and positive perceptions of internal strengths leading to higher motivation to preserve and maintain resilience in late life [66]. By using the SLSAS measure with older clients, counseling psychologists may be able to assist clients to develop a realistic self profile of their sources of life strengths in relation to self-growth and control over every day life situation. Conversely, counseling psychologists' own understanding of their older clients' perceived sources of strengths may serve as a basis for encouraging clients to develop new areas of strength and, where relevant, to draw on internal sources of strengths such as reflecting on meaning and purpose for life, maintaining social ties, and finding satisfaction through continuing pursuit of earlier life goals and plans [32]. Counselors may be able to help clients develop existing sources of life strengths by reflecting on prior experience of having overcome negative conditions, and thereby seeking to harness the positive potentials (see [67]). The SLSAS also has considerable potential for predicting psychological resilience if used with middle-aged individuals who are concerned about the prospects for successful aging in their later years.

### 7.3. Limitations of the Present Research

The sample in the current study was predominantly Caucasian and this factor may limit the generalizability of the findings. As is the case with most contemporary scientific approaches, the theoretical and measurement models we employed in the development of the SLSAS measure are, by and large, embedded within a Western culture that places an emphasis on individualistic and personal values such as personal achievement, self-efficacy, and self-directed competence (see [68]). It is reasonable to assume that in a non-Western collectivistic sample, a sense of community relatedness and personal meaning and belongingness may figure more predominantly than appears to be the case in our present Caucasian sample. Earlier research in a number of other contexts (see [67]) has indicated that people in strong collectivistic cultures may be more concerned about securing a better life for their family than for themselves. Also their life satisfactions may come more from interpersonal harmonious relationships, group morale, and collaborative success as contrasted with a predominance of intrapersonal factors such as factors of self-reliance, financial independence and self-care activities which were important to the Caucasian samples we used in the present study. It now remains for future cross-cultural researchers to compare the SLSAS factorial structures with those derived from the responses of older adults from non-Caucasian backgrounds in order to establish further the universality of the “sources of life strengths” construct(s). Suffice it to say that the SLSAS has the potential to stimulate further research along a number of other cross-cultural dimensions.

Another issue that needs to be addressed in future studies of the SLSAS has to do with the potential of the SLSAS to predict resilience when conceptualized within a process-oriented framework, as distinguished from an intrapersonal framework. In our present delineation and definition of “life strengths” and “resilience,” our conceptualization of both of these constructs was that they involve a combination of intrapersonal and interpersonal processes. However, both scales of resilience [15, 16] that we used against which to establish the concurrent validity of the SLSAS viewed resilience to be a stable dispositional intrapersonal trait and not an ongoing process. Unfortunately, within the time constraints of our study and the predetermined scope of our research (where the primary goal was to construct a scale for appraising sources of life strengths with the potential to predict resilience) we were obliged, at the time of the 24-month followup, to use the best available standardized scales of resilience. Future research using the SLSAS should consider its potential to predict resilience as being more process-oriented rather than a dispositional intrapersonal trait.

## Figures and Tables

**Table 1:** Factor structure of the SLSAS for exploratory factorial analysis (EFA) with samples one and two (*N* = 240) combined and principal component analysis (PCA) with sample three (*N* = 190).

SLSAS scale items^a^	I		II		III		IV		V		VI		VII		VIII		IX		X		Factor VIII Factor IX
										Factor loadings^b^											
	EFA	PCA	EFA	PCA	EFA	PCA	EFA	PCA	EFA	PCA	EFA	PCA	EFA	PCA	EFA	PCA	EFA	PCA	EFA	PFA	
Personal self-efficacy beliefs																					
Ability to overcome problems	.85	.79																			
Ability to succeed	.76	.74																			
High motivation to excel	.72	.77																			
Excellent knowledge	.77	.82																			
Ability to obtain information	.69	.67																			
Personal maturity																					
Strong meaning for life			.78	.82																	
Awareness of limitations			.82	.79																	
Well-defined goals	.47		.61	.51																	
Desire for spirituality			.70	.73																	
Accepting of negative consequences			.79	.75																	
Personal traits																					
Courageous					.46	.50															
Truthfulness and honesty					.75	.75															
Autonomous and self-reliant					.81	.81															
Responsible					.59	.56															
Faithful and loyal					.79	.77															
Self-care routines																					
Diligent							.81	.76													
Follow sound diet regimen							.69	.69													
Persistent in task completion							.79	.75													
Exercise regularly							.69	.77													
Devotional prayer regularly							.70	.76													
Personal commitments																					
Desire to make a positive social impact									.77	.75											
Committed to personal mission									.70	.72											
Committed to leaving legacy									.70	.70											
Committed to life of service									.79	.77											
Committed to imparting education to the young									.69	.63											
Personal achievements																					
Achieved important career/life goals											.61	.48									
Achieved social success											.49										
Achieved family respect											.60	.54									
Achieved financial success											.49										
Achieved outside (external) recognition											.47	.42									
Attitudes toward Life																					
Optimistic and hopeful for future													.72	.78							
Life is a challenge	.46												.69	.72							
Life is an adventure													.73	.74							
Believe there is solution to all problems													.81	.74							
Believe human nature is good													.88	.80							
Social support: informal sources																					
Intimate relationships															.88	.83					
Friends and associates															.75	.72					
Formal social service agency															.69	.66					
Seniors' associations and clubs															.70	.69					
Religious institutions															.72	.71					
Monetary status																					
Having sound investments																	.81	.87			
Having stable credit rating																	.69	.71			
Having good prospects for inheritance																	.72	.69			
Having steady income from pension																	.71	.75			
Having means for more earnings																	.69	.62			
Continuity in mid-life roles																					
Opportunity to continue in mid-life careers and positions																			.74	.72	
Continuing opportunity for achieving recognition and awards for past achievements																			.90	.87	
Continuing opportunity to be involved in parenting function																			.79	.79	
Continuing opportunity to pursue mid-life hobbies/interest.																			.73	.77	
Continuing opportunity to pursue mid-life friendships																			.81	.87	

^a^SLSAS items are identified by key words in item. ^b^All loadings > 0.40 are shown.

EFA was conducted on 60 items: ten items were subsequently dropped because of unacceptably high item-total correlations above 0.80. The first component had an explained variance of 38% followed by five more components accounting for a cumulative variance of 20% and the last four providing a cumulative variance of 12% (total percent of explained variance is 70).

PCA was conducted on 50 items: the first component had an explained variance of 32% followed by five more accounting for a cumulative variance of 23% and the last four providing a cumulative variance of 15% (total percent of explained variance = 70).

Note: Table 1 shows factor loadings for only those 50 items that were used in both the EFA and PCA.

**Table 2:** Intercorrelations between subscale factors of the SLSAS (*N* = 430).

Subscales	(1)	(2)	(3)	(4)	(5)	(6)	(7)	(8)	(9)	(10)
(1) Personal efficacy	—	.24**	.20**	.25**	.21**	.18*	.27**	−.12*	−.24**	.25**
(2) Personal maturity		—	.22**	.34**	.16	.18	.21**	.21*	−.16	−.19*
(3) Personal traits			—	.25**	.17	.21*	.26**	−.15	−.21*	.14
(4) Self-care routines				—	.23**	.24**	.13	.19*	.24**	−.20*
(5) Personal commitments					—	−.12	.17	.27**	−.12	.26**
(6) Personal achievements						—	.27**	.14	.22*	.27**
(7) Attitudes toward life							—	.17	.16	.13
(8) Social support: informal								—	.15	.17
(9) Monetary status									—	.28**
(10) Continuity in mid-life roles										—

*P < 0.05; **P < 0.001.

**Table 3:** Correlations of SLSAS scales with measures of internal control, self-esteem, life satisfaction, vulnerability, and depression.

	Internal control	Self-esteem	Life satisfaction	Vulnerability	Depression
SLSAS Scales					
Personal self-efficacy beliefs	.65***	.69***	.48***	−.48***	−.50***
Personal maturity	.41***	.26**	.38***	−.49***	−.33**
Personal traits	.33**	.35**	.29**	−.22*	−.21*
Self-care routines	.21*	.24**	.22*	.14	−.29**
Personal commitments	.59***	.57***	.52***	−.36**	−.49***
Attitudes toward life	.46**	.54***	.37**	−.45***	−.31**
Personal achievements	.37**	.36**	.31**	−.42**	−.1.4
Social supports: informal sources	* * −.21*	* * −.34**	.12	−.08	−.18
Monetary status	.12	.20*	.48***	−.27*	−.24*
Continuity in mid-life roles	.28**	.58***	.48***	−.32**	−.26*

High scores indicate high levels of personal control, self-esteem, life satisfaction, vulnerability, and depression.

*P < 0.05; **P < 0.01; ***P < 0.001.

**Table 4:** Correlation coefficients of SLSAM scales with outcome measures of global resilience and its four subscales of perceived challenge, controlled processing, commitment to living (DRS: dispositional resilience scale, Bartone et al., 1989 [15]) administered at 24-month followup from date of baseline assessments of the SLSAS measure (sample = 150).

	* *Global	Perceived challenge	Controlled processing	Commitment to living
SLSAS Scale				
Personal self-efficacy beliefs	.51***	.56***	.53***	.67***
Personal maturity	.31**	.29**	.26**	.31**
Personal traits	.39***	.43***	.29*	.27**
Self-care routines	.21*	.21*	.15	.29**
Personal commitments	.49***	.47***	.46***	.51***
Attitudes toward life	.46**	.54***	.45***	.31**
Personal achievements	.17	.16	.21*	.16
Social supports: informal sources	.21*	.23*	.22*	.25*
Monetary status	.22*	.26*	.22*	.20*
Continuity in mid-life roles	.28*	.46***	.38***	.40***

High scores indicate high global resilience, high level of perceived challenge, high level of controlled processing, and high level of commitment to living.

*P < 0.05; **P < 0.01; ***P < 0.001.

**Table 5:** Correlation coefficients of SLSAS scales with outcome measures of total resilience, perseverance, equanimity, meaningfulness, self-reliance, and existential aloneness administered at 24-month followup from date of baseline assessment (sample = 150).

	Total resilience	Perseverance	Equanimity	Meaningfulness	Self-reliance	Existential aloneness
SLSAS Scale						
Personal self-efficacy beliefs	.55***	* *.69***	* *.52***	.51***	* *.34***	* *.43***
Personal maturity	.41***	* *.29**	* *.38***	.46***	* *.43**	* *.36**
Personal traits	.38***	* *.41***	* *.29**	.23*	* *.21*	* *.41***
Self-care routines	.21*	* *.24*	* *.22*	.14	* *.39**	* *.21*
Personal commitments	.59***	* *.57***	* *.52***	.36***	* *.59***	* *.32**
Attitudes toward life	.46**	* *.54***	* *.37***	.45***	* *.31**	* *.28*
Personal achievements	.31**	* *.16	* *.31**	.22*	* *.34**	* *.37**
Social supports: informal sources	.21*	* *.24*	* *.12	.08	* *.18	* *.21*
Monetary status	.32**	* *.20*	* *.22*	.27*	* *.44***	* *.48***
Continuity in mid-life roles	.48***	* *.56***	* *.46***	.22*	* *.46***	* *.32**

High scores indicate high levels of total resilience; perseverance; personal equanimity; meaningfulness; self-reliance; existential aloneness.

*P* < 0.05;**P < 0.01; ***P < 0.001.

## Appendix

Specimen items for nine structures of the SLSAS measure, each structure carrying five items,

I believe my strength to overcome problems and to sustain my present sense of well-being comes from (each rated* very important  =  5*—*not at all important  =  1*):

structure one items:

- from my beliefs and self-confidence that I can continue to succeed in the future,
- from my very good understanding of the people around me;

structure two items:

- from my strong sense of meaning and purpose for my life,
- from my having well-defined goals and plans for living;

structure three items:

- from my desire to be independent and self-reliant under most circumstances,
- from my desire to be faithful and honest in most of my life's functions and roles;

structure four items:

- from my habit of engaging in regular devotional prayer and worship,
- from my strong desire to be regular and persevering in most tasks assigned to me;

structure five items:

- from my desire to make a positive social impact on people around me,
- from my commitment to lead a life of service to others;

structure six items:

- from my optimistic and cheerful attitude to life,
- from the fact that I see life as being a pleasurable adventure;

structure seven items:

- from the knowledge that I have a circle of intimate friends and relationships,
- from my association with social net works who have high moral and religious principles;

structure eight items:

- from the knowledge that I have a solid income source,
- from the knowledge that I am not, nor need to be, financially dependent on anyone else;

structure nine items:

- from the opportunities I have to continue with my life goals, roles, and activities from my earlier years of life,
- from continuing opportunities to gain and receive respect and acknowledgment from others for contributions I made during my earlier years of life.

## References

[1] Seligman ME, Csikszentmihalyi M (2000). Positive psychology. An introduction. *The American Psychologist*.

[2] Aspinwall LG, Staudinger UM (2003). *A Psychology of Strengths: Fundamental Questions and Future Directions for a Positive Psychology*.

[3] Brandtstädter J, Wentura D, Rothermund K, Brandtstädter J, Lerner RM (1999). Intentional self-development through adulthood and later life: tenacious pursuit and flexible adjustment of goals. *Action and Self-Development: Theory and Research Through the Life-Span*.

[4] Bandura A (1997). *Self-Efficacy: The Exercise of Control*.

[5] Fry PS, Debats DL, Fry PS, Keyes CLM (2010). Sources of human life-strengths, resilience, and health. *New Frontiers in Resilient Aging: Life Strengths and Well-Being in Late Life*.

[6] Chapin R (1995). Social policy development: the strength perspective. *Social Work*.

[7] Chapin R, Nelson-Becker H, MacMillion K, Berkman B (2006). Strength- based and solution-focused approaches to practice. *Handbook of Social Work in Health and Aging*.

[8] Schlegel RJ, Hicks JA (2011). The true self and psychological health: emerging evidence and future directions. *Social and Personality Psychology Compass*.

[9] Ungar M (2012). *The Social Ecology of Resilience*.

[10] Maslow AH (1968). *Toward a Psychology of Being*.

[11] Saleeby D (1997). *The Strength Perspective in Social Work Practice*.

[12] Kivnick HQ, Pollock GH, Greenspan SJ (1998). Through the life cycle: psychological thoughts on old age. *The Course of Life*.

[13] Hobfoll SE (2002). Social and psychological resources and adaptation. *Review of General Psychology*.

[14] Vaillant G (2002). *Aging Well*.

[15] Bartone PT, Ursano RJ, Wright KM, Ingraham LH (1989). The impact of a military air disaster on the health of assistance workers: a prospective study. *Journal of Nervous and Mental Disease*.

[16] Wagnild GM, Young HM (1993). Development and psychometric evaluation of the Resilience Scale. *Journal of Nursing Measurement*.

[17] Baumeister RF (1991). *Meanings of Life*.

[18] Fry PS (2001). The unique contribution of key existential factors to the prediction of psychological well-being of older adults following spousal loss. *Gerontologist*.

[19] Fry PS (2000). Religious involvement, spirituality and personal meaning for life: existential predictors of psychological wellbeing in community-residing and institutional care elders. *Aging and Mental Health*.

[20] Steger MA, Oishi S, Kashdan TB (2009). Meaning in life across the life span: levels and correlates of meaning in life from emerging adulthood to older adulthood. *Journal of Positive Psychology*.

[21] Hoffman L, Hoffman L, Yang M, Kaklauskas FJ, Chan A (2009). Introduction to existential psychology in a cross-cultural context: an east-west dialogue. *Existential Psychology East-West*.

[22] Debats DL, Wong PTP, Fry PS (1998). Measurement of personal meaning: the psychometric properties of the life regard index. *The Human Quest for Meaning: A Handbook of Psychological Research and Clinical Applications*.

[23] Reker GT, Woo LC (2011). Personal meaning orientations and psychosocial adaptation in older adults. *SAGE Open*.

[24] Fry PS (2001). Predictors of health-related quality of life perspectives, self-esteem, and life satisfactions of older adults following spousal loss: an 18-month follow-up study of widows and widowers. *Gerontologist*.

[25] Fry PS (2003). Perceived self-efficacy domains as predictors of fear of the unknown and fear of dying among older adults. *Psychology and Aging*.

[26] Fry PS, Debats DL (2003). Domain-specific social comparison orientations as predictors of health-related quality of life, life satisfaction, and general optimism in late life functioning. *Journal of Mental Health and Aging*.

[27] McAvay GJ, Seeman TE, Rodin J (1996). A longitudinal study of change in domain-specific self-efficacy among older adults. *Journals of Gerontology B*.

[28] Featherman DL, Smith J, Peterson JG, Baltes PB, Baltes MM (1990). Successful aging in a post-retired society. *Successful Aging: Perspectives from the Behavioral Sciences*.

[29] Rowe JW, Kahn RL (1987). Human aging: usual and successful. *Science*.

[30] Ryff CD, Singer B, Bengston VL, Gans D, Pulney NM, Silverstein M (2009). Understanding healthy aging: key components and their integration. *Handbook of Theories of Aging*.

[31] Baltes PB, Baltes MM, Baltes PB, Baltes MM (1990). Psychological perspectives on successful aging: the model of selective optimization with compensation. *Successful Aging: Perspectives from the Behavioral Sciences*.

[32] Atchley RC (1999). *Continuity and Adaptation in Aging*.

[33] Davydov DM, Stewart R, Ritchie K, Chaudieu I (2010). Resilience and mental health. *Clinical Psychology Review*.

[34] Keyes CL, Lopez SJ, Snyder CR, Lopez SJ (2002). Toward a science of mental health: positive directions in diagnosis and interventions. *Handbook of Positive Psychology*.

[35] Staudinger UM, Marsiske M, Baltes PB, Cicchetti D, Cohen DJ (1995). Resilience and reserve capacity in later adulthood: potentials and limits of development across the life span. *Developmental Psychopathology: Vol. 2, Risk, Disorder, and Adaptation*.

[36] Davidson RJ, Jackson DC, Kalin NH (2000). Emotion, plasticity, context, and regulation: perspectives from affective neuroscience. *Psychological Bulletin*.

[37] Waterman AS (2007). On the importance of distinguishing hedonia and eudaimonia when contemplating the hedonic treadmill. *American Psychologist*.

[38] Ungar M, Lemer RM (2008). Introduction to a special issue of research on human development: resilience and positive development across the life span: a view of the issues. *Research in Human Development*.

[39] Ungar M, Liebenberg L (2009). Cross-cultural consultation leading to the development of a valid measure of youth resilience: the international resilience project. *Studia Psychologica*.

[40] Canadian Index of Well-being Measuring what matters.

[41] Huta V, Ryan RM (2010). Pursuing pleasure or virtue: the differential and overlapping well-being benefits of hedonic and eudaimonic motives. *Journal of Happiness Studies*.

[42] Martin P (2002). Individual and social resources predicting well-being and functioning in later years: conceptual models, research, and practice. *Ageing International*.

[43] Ong AD, Bergeman CS (2004). Resilience and adaptation to stress in later life: empirical perspectives and conceptual implications. *Ageing International*.

[44] Sherer M, Maddux RW, Mercandante B, Prentice-Duce S, Jacobs B, Rogers RW (1982). The self-efficacy scale: construction and validation. *Psychological Reports*.

[45] Lachman ME, Weaver SL (1997). The sense of control as a moderator of social class differences in health and well-being. *Journal of Personality and Social Psychology*.

[46] Antonucci TC, Birren JE, Schaie KW (2001). Social relations: an examination of social networks, social support and sense of control. *Handbook of the Psychology of Aging*.

[47] Rosenberg M, Schooler C, Schoenbach C, Rosenberg F (1995). Global self-esteem and specific self-esteem: different concepts, different outcomes. *American Sociological Review*.

[48] Diener E, Emmons RA, Larsen RJ, Griffin S (1985). The satisfaction with life scale. *Journal of Personality Assessment*.

[49] Schofield MJ, Reynolds R, Mishra GD, Powers JR, Dobson AJ (2002). Screening for vulnerability to abuse among older women: women’s Health Australia Study. *Journal of Applied Gerontology*.

[50] Zung WW (1965). A self-rating depression scale. *Archives of General Psychiatry*.

[51] Maddi SR, Kobasa SC (1984). *The Hardy Executive: Health Under Stress*.

[52] Travis SS, Bernard MA, McAuley WJ, Thornton M, Kole T (2003). Development of the family caregiver medication administration hassles scale. *Gerontologist*.

[53] Keyes CLM, Shmotkin D, Ryff CD (2002). Optimizing well-being: the empirical encounter of two traditions. *Journal of Personality and Social Psychology*.

[54] Baumeister RF, Leary MR (1995). The need to belong: desire for interpersonal attachments as a fundamental human motivation. *Psychological Bulletin*.

[55] Kaiser HF (1970). A second generation little jiffy. *Psychometrika*.

[56] Kaiser HF (1974). An index of factorial simplicity. *Psychometrika*.

[57] Stevens J (1996). *Applied Multivariate Statistics for the Social Sciences*.

[58] Cattell RB (1966). The scree test for the number of factors. *Multivariate Behavioral Research*.

[59] Clark LA, Watson D (1995). Constructing validity: basic issues in objective scale development. *Psychological Assessment*.

[60] Pollock SE, Duffy ME (1990). The health-related hardiness scale: development and psychometric analysis. *Nursing Research*.

[61] Nunnally JC (1978). *Psychometric Theory*.

[62] Streiner DL, Norman GR (1995). *Health Management Scales: A Practical Guide to their Development and Use*.

[63] Miller DC (1991). *Handbook of Research Design and Social Measurement*.

[64] Lawton MP, Moss M, Hoffman C, Grant R, Have TT, Kleban MH (1999). Health, valuation of life, and the wish to live. *Gerontologist*.

[65] McGee DL, Liao Y, Cao G, Cooper RS (1999). Self-reported health status and mortality in a multiethnic cohort. *American Journal of Epidemiology*.

[66] Schlegel RJ, Hicks JA, King LA, Arndt J (2011). Feeling like you know who you are: perceived true self-knowledge and meaning in life. *Personality and Social Psychology Bulletin*.

[67] Wong PTP (2011). Positive psychology 2.0: towards a balanced interactive model of the good life. *Canadian Psychology*.

[68] Schwartz SH, Bilsky W (1990). Toward a theory of the universal content and structure of values: extensions and cross-cultural replications. *Journal of Personality and Social Psychology*.

